# Reactive and pre-emptive vaccination strategies to control hepatitis E infection in emergency and refugee settings: A modelling study

**DOI:** 10.1371/journal.pntd.0006807

**Published:** 2018-09-25

**Authors:** Ben S. Cooper, Lisa J. White, Ruby Siddiqui

**Affiliations:** 1 Mahidol Oxford Tropical Medicine Research Unit (MORU), Bangkok, Thailand; 2 Centre for Tropical Medicine and Global Health, Nuffield Department of Clinical Medicine, University of Oxford, Oxford, United Kingdom; 3 Médecins Sans Frontières-UK, London, United Kingdom; Johns Hopkins Bloomberg School of Public Health, UNITED STATES

## Abstract

**Background:**

Hepatitis E Virus (HEV) is the leading cause of acute viral hepatitis globally. Symptomatic infection is associated with case fatality rates of ~20% in pregnant women and it is estimated to account for ~10,000 annual pregnancy-related deaths in southern Asia alone. Recently, large and well-documented outbreaks with high mortality have occurred in displaced population camps in Sudan, Uganda and South Sudan. However, the epidemiology of HEV is poorly defined, and the value of different immunisation strategies in outbreak settings uncertain. We aimed to estimate the critical epidemiological parameters for HEV and to evaluate the potential impact of both reactive vaccination (initiated in response to an epidemic) and pre-emptive vaccination.

**Methods:**

We analysed data from one of the world's largest recorded HEV epidemics, which occurred in internally-displaced persons camps in Uganda (2007–2009), using transmission dynamic models to estimate epidemiological parameters and assess the potential impact of reactive and pre-emptive vaccination strategies.

**Results:**

Under baseline assumptions we estimated the basic reproduction number of HEV in three separate camps to range from 3.7 (95% Credible Interval [CrI] 2.8, 5.1) to 8.5 (5.3, 11.4). Mean latent and infectious periods were estimated to be 34 (95% CrI 28, 39) and 40 (95% CrI 23, 71) days respectively.

Assuming 90% vaccine coverage, reactive two-dose vaccination of those aged 16–65 years excluding pregnant women (for whom vaccine is not licensed), if initiated after 50 reported cases, led to mean camp-specific reductions in mortality of 10 to 29%. Pre-emptive vaccination with two doses reduced mortality by 35 to 65%. Both strategies were more effective if coverage was extended to groups for whom the vaccine is not currently licensed. For example, two dose pre-emptive vaccination, if extended to include pregnant women, led to mean reductions in mortality of 66 to 82%.

**Conclusions:**

HEV has a high transmission potential in displaced population settings. Substantial reductions in mortality through vaccination are expected, even if used reactively. There is potential for greater impact if vaccine safety and effectiveness can be established in pregnant women.

## Author summary

Hepatitis E virus is a leading cause of acute viral hepatitis in developing countries. About 20% of those infected develop clinical symptoms; of those, about 2% of non-pregnant cases and 20% of pregnant cases die. There is a safe and effective HEV vaccine that is licensed in China for those aged 16–65 years who are not pregnant. The potential for using this vaccine in outbreak settings has not previously been examined. We analysed data from one of the world’s largest recorded HEV epidemics. We estimated that one case infects, on average, between 4 and 9 others at the start of an epidemic. We found that vaccination restricted to those aged 16–65 who are not pregnant could reduce mortality in outbreak settings by between about 10 and 30% if used reactively (initiating vaccination after the start of an epidemic); pre-emptive vaccination of the same group could reduce mortality by 35–65%. Substantially higher reductions in mortality are likely if vaccination can be safely extended to pregnant women and other age groups without loss of effectiveness. However, even if this is possible, reactive vaccination is unlikely to reduce mortality by more than 50% while pre-emptive vaccination can reduce mortality by 80 to 100%.

## Introduction

Communicable diseases are responsible for large excess mortality and morbidity in complex emergencies [1]. Epidemic Hepatitis E is a particular concern due to high mortality in pregnant women and lack of interventions of proven effectiveness in emergency settings [2]. Hepatitis E is caused by a single-stranded RNA virus from the Hepeviridae family. There is one serotype but four genotypes [3]. Hepatitis E virus (HEV) is enterically-transmitted and has caused large outbreaks in many regions including China, the Indian subcontinent, central Asia and East Africa [4]; globally, it is a leading cause of acute viral hepatitis and has been estimated to account for ~10,000 annual pregnancy-related deaths in southern Asia alone [5]. Genotypes 1 and 2 dominate in Africa and most of Asia and are found only in humans; they have been estimated to cause over 20 million infections annually in these regions [6]. Recently, large outbreaks associated with high mortality have occurred in internally-displaced persons (IDP) camps in Sudan, Uganda and South Sudan [7–10].

The risk of symptomatic illness given HEV infection is thought to increase with age and has been estimated to be about 20% in adults infected with genotypes 1 or 2 [6]; the probability of death given symptomatic infection has been estimated to be approximately 2% for non-pregnant cases and 20% in pregnant women [6,11].

With over 60 million people living as refugees or otherwise forcibly displaced worldwide [12], the majority in areas vulnerable to HEV infection, understanding HEV epidemiology and the potential for its control is of considerable importance.

### Course of hepatitis E infection

Knowledge of the course of HEV infection is based mainly on two volunteer studies [13,14] and three patient studies [15–17]. The two single volunteer studies (with known dates of exposure) gave incubation periods of 38 and 39 days respectively [13,14]. Clinical illness typically lasts 1–4 weeks [17]. Viral shedding in the stool begins about four to five weeks after infection and continues for up to four to five weeks [13,14,18–20]. The start of the infectious period appears to approximately coincide with the onset of the prodromal phase of disease (i.e. early non-specific symptoms) [14].

Transmission of HEV is widely thought to occur predominantly via the faecal-oral route, usually through contact with contaminated water; it might therefore be assumed that household person-to-person transmission is rare. However, a case-control study of 112 symptomatic cases and 145 controls in Paloga, Uganda found only two behavioural risks associated with symptomatic HEV infection (with adjusted odds ratios of 3 and 2 respectively): use of wide-mouthed water storage vessels and communal hand washing [21]. Drinking water from the river and having a borehole as a primary source of drinking water were not associated with HEV risk. Based on these findings, and the presence of HEV RNA in hand-rinse samples (but not in any of the 15 drinking water samples collected), the authors concluded that water storage practices could have played an important role and that transmission was likely to have included household-level or person-to-person spread. A study during a large HEV outbreak in Madi Opei, Uganda reached a similar conclusion, and reported multiple lines of evidence to suggest that person-to-person household transmission of HEV contributed to the epidemic, and was unable to detect HEV in drinking water or zoonotic sources [22].

### Vaccine

There is a recombinant vaccine, HEV 239, which is approved for use in China in those aged 16–65 years who are not pregnant [23]. It is produced with a genotype 1 isolate and efficacy against both genotypes 1 and 4 has been established in non-human primates. The vaccine has been demonstrated to have >90% efficacy with a three dose schedule (0, 1 and 6 months) based on a clinical trial involving 109,959 people at risk of HEV infection in an endemic setting, primarily with genotype 4 [23]. Preliminary observations suggest the vaccine is also safe and effective in pregnant women [24]. Clinical trial data are lacking for those aged <16 or >65 years, in areas where genotypes 1 and 2 dominate, and in outbreak settings.

We aimed to quantify key epidemiological parameters for HEV in IDP camp settings and evaluate the potential benefits of vaccination. We consider both pre-emptive (prior to HEV cases occurring) and reactive vaccination (once HEV outbreaks are already underway), and evaluate the potential impact of selecting different target groups to receive the vaccine.

To do this we fitted dynamic transmission models to data from three large HEV outbreaks in IDP camps. We used a Bayesian framework to combine data from previous studies with observed epidemic data to obtain an improved understanding of the natural history of HEV infection, quantify the transmission potential, and evaluate the potential for vaccination to reduce the number of clinical cases and associated mortality.

## Methods

### Data

Data came from three outbreaks in 2007–2009 from IDP camps in the district of Kitgum, Uganda: Agoro, Madi Opei, and Paloga (estimated populations 16,689, 10,442, and 10,555 respectively). These outbreaks were part of a larger epidemic in Kitgum district. Conditions in the camps were crowded and in all camps access to water and sanitation was initially poor [21]. Though the camps were in close proximity, they were not within easy walking distance of each other and there was little population movement between camps. Jaundice cases were recorded in facility-based passive surveillance systems, with all suspected cases referred to the MSF clinic at Madi Opei. Evidence from serology and reverse transcription–PCR confirmed HEV genotype 1 to be the outbreak cause; other causes of viral hepatitis were rare [10]. All patient data used in this study were anonymized.

### Transmission model

**Fig 1:**
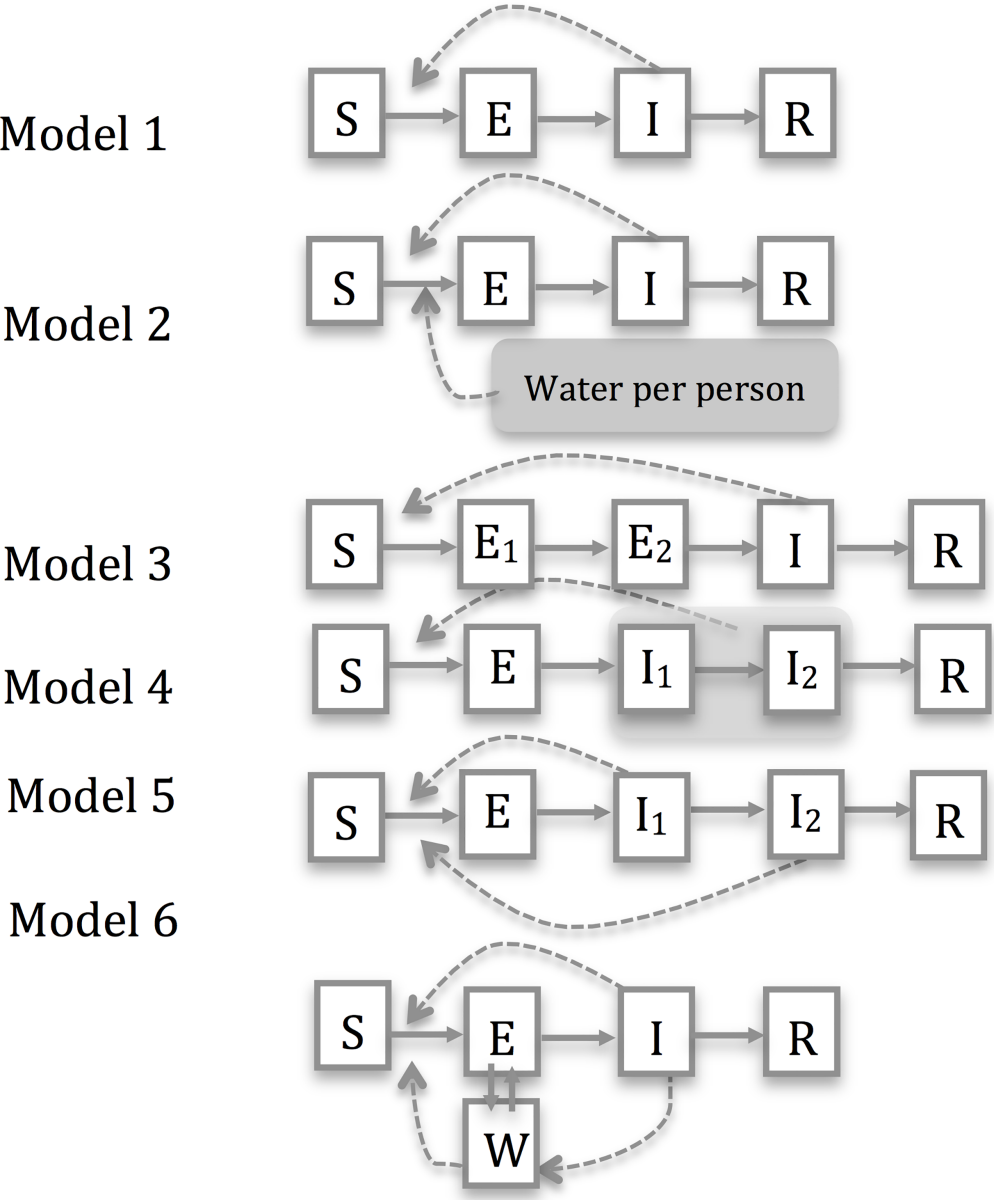
Flow diagrams for the models considered in the analysis. Model compartments are indicated by boxed letters and transitions between compartments are shown by solid lines. Broken lines indicate how variables affect transition rates. In our baseline model (Model 1) individuals were assumed to be in one for four possible states: susceptible to infection (S); latently infected but not yet infectious (E); infectious (I); and recovered and immune (R). Extensions of this model are made to: i) allow for rates of patient-to-patient transmission to be affected by the water and sanitation intervention, specified by external data giving either the presence/absence of the intervention at each time point or water sources (taps) per person (Model 2, S1 Fig); ii) to allow for different implicit distributions for time periods in latent and infectious compartments (Models 3–5, where in model 5 we allow for different rates of transmission from patients in early and late infectious compartments, I_1_ and I_2_); iii) to allow for the existence of a saturating environmental reservoir (Model 6). Extending these models to account for vaccination would correspond to adding a new solid arrow from the *S* to the *R* compartment in each of the models (vaccination is assumed to have no impact on those already infected).

We fit a series of deterministic transmission models to the data. We assumed latent and infectious periods and the probability of infections being reported were common to all camps, as the demographics and provision of healthcare at the three camps was similar.We allowed transmissibility to vary by camp, as this might be expected to depend on local camp conditions (Fig 1). In our baseline model (Model 1) individuals were assumed to be in one of four possible states: susceptible to infection (S); latently-infected but not yet infectious (E); infectious (I); and recovered and immune (R). The rate at which susceptibles became infected was assumed to scale linearly with the number currently infectious. Information from previous studies was used to construct informative prior distributions (priors) for natural history parameters. These priors represent knowledge about disease progression parameters before fitting the model to the outbreak data. When combined with analysis of the data they give rise to posterior distributions, which represent what we know about the parameters after analysing the new data.

A human challenge study had previously reported that viable virus could be found in the faeces four days before onset of the icteric phase of disease (i.e. jaundice) [14]. We therefore assumed that patients become infectious one week before the icteric phase of disease. A prior for the mean infectious period was derived from a study of faecal shedding in 11 patients with sporadic acute HEV infection acquired in Bangladesh, Vietnam, Nepal, and Japan (Table 1) [20]; this study found that viable HEV could be recovered from faecal samples up until 2–5 weeks after hepatitis onset. We also performed sensitivity analyses where we based this prior on duration of faecal shedding from the single patient with HEV genotype 1 in this study. The prior distribution for the latent period was based on a single observation from the same study where there was a delay of 34 days between inoculation and viable HEV in faeces. Faecal shedding of HEV in asymptomatically infected people is known to occur [25]; we assumed no difference in faecal viral shedding between symptomatic and asymptomatic individuals. We used seroprevalence data to derive an informative prior for the proportion of infections that are reported (Table 1). By default we assumed no immunity to HEV in the IDP camp populations prior to first reported case, consistent with the absence of reports of previous HEV epidemics in Kitgum district and serological data [10]. We performed multiple sensitivity analyses, considering models with: i) a different prior for the infectious period; ii) camp-specific transmission rates affected by a water and sanitation intervention (Models 2a and 2b); iii) different assumptions about the distribution of the latent and infectious periods (Models 3–5); and iv) allowing for between 10% and 30% of the population to be initially immune to HEV infection, prior to the start of the outbreak. All the above models assumed nothing about the relative importance of different modes of transmission and are consistent with both transmission mediated by a contaminated environment (though without long-term virus persistence in this environment) and direct person-to-person spread. We also compared our findings with those from a model (Model 6) explicitly accounting for a second mode of transmission, an unobserved environmental reservoir of HEV where virus may persist for longer time periods. We performed an extensive sensitivity analysis by running this model under 25 different prior assumptions representing combinations of five different assumptions about the relative importance of this environmental reservoir in the early stages of an epidemic and five different assumptions about persistence of viable virus in the environment.

**Table 1:** Model priors.

Parameter^1^	Models	Prior	Notes
Basic reproduction number, *R*_*0*_	Model 1	Uniform(1,100)	No previous estimates were available to inform a prior, though we can be sure that *R*_*0*_ >1. In Model 1 *R*_*0*_ was given an explicit prior and the transmission parameter, *β*, was derived. In other models *β* was given an explicit prior and *R*_*0*_ derived.
Transmission parameter, *β*	Models 2–6	Uniform(0.0001,500)	Very similar results were obtained whether using a uniform distribution or a diffuse Gamma distribution with shape and rate 0.001.
Rate of leaving latent compartment,γ	All except Model 3 Model 3	Gamma with shape = 1 rate = 34/365 Gamma with shape = 2 rate = 34/365	Informative prior derived using the fact that the Gamma distribution is a conjugate prior for an exponential model. It is informed by the single observation of a latent period of 34 days (from Chauhan *et al*, 1993 [14]). In Model 3 the shape was doubled to account for the fact that the latent period was represented using two compartments.
Rate of leaving infectious compartment, *ρ*	All except Models 1b, 4 and 5 Model 1b Models 4 and 5	Gamma with shape = 11 rate = 11 × 38/365 Gamma with shape = 1 rate = 11 × 29/365 Gamma with shape = 22 rate = 11 × 38/365	Informative prior derived using the fact that the Gamma distribution is a conjugate prior for an exponential model. It is informed by data from Takahashi et al (2007)[20] where the duration of faecal shedding in 11 patients was observed. Assuming individuals are infectious one week before the onset of the icteric phase of disease the mean duration of shedding was 38 days. In Model 1b only the data from the single patient with HEV genotype 1 in Takahashi et al is used [20] In Models 4 and 5 the shape was doubled to account for the fact that infectious period was represented by two compartments.
Proportion of people who are infected who are reported as cases, π	All	Beta(2, 8)	The prior chosen corresponds to the posterior that would be obtained using Beta-binomial conjugacy if 1 out of 8 people infected were known to have been reported as cases (starting with a vague Beta(1,1) prior). This was informed by a seroprevalence study at Madi Opei towards the end of the outbreak where, assuming no prior immunity, about 1 in 7.5 of those infected were reported as cases [26]. Note that this parameter is used when calculating the threshold for reactive vaccination, and will, in general, be lower than the probability of symptoms given infection.
Negative binomial dispersion parameter (see model fitting section in Methods)	All	Uniform(0.1,10000)	No prior information
Time of first infection	All	Uniform (but constrained to be before the first case)	No prior information
Rate of contamination of saturating environment per infected host, *λ*	Model 6	Uniform(0.0001,1)	No information, but for values higher than 1 saturation is too fast to be plausible
Rate of loss of contamination from environmental reservoir, υ	Model 6	w1: Gamma with shape = 5.245, scale = 34.795 w2: Gamma with shape = 3.663, scale = 14.234 w3: Gamma with shape = 6.837, scale = 3.813 w4: Gamma with shape = 51.296, scale = 0.254 w5: Gamma with shape = 51.296, scale = 0.127	A wide range of priors were considered, consistent with observations of persistence of HEV in soil [27]. Prior w1 corresponds to a mean persistence in the environment of 2 days (95% equal-tailed interval:(1, 6)); w2 corresponds to 7 days (3, 28); w3 corresponds to 14 days (7.5, 35.3) days; w4 corresponds to 28 (21.7, 37.6) days; w5 corresponds to 56 (43.3, 75.1) days.
Initial proportion of transmission that occurs via contamination of the saturating environmental reservoir	Model 6	p1: Beta(4.24, 80.51) p2: Beta(7.93, 23.79) p3: Beta(20.61, 20.61) p4: Beta(23.79, 7.93) p5: Beta(80.51, 4.24)	Prior p1 corresponds to a mean (95% equal-tailed interval) of 0.05 (0.015, 0.105); p2 to 0.25 (0.12, 0.41); p3 to 0.5 (0.35, 0.65); p4 to 0.75 (0.59,0.88); p5 to 0.95 (0.90, 1.00).
Proportion of transmission occurring in the first part of the infectious period	Model 5	Beta(1,1)	Non-informative prior reflects lack of information.

^**1**^ Time units are in years.

### Model fitting

Model fitting was performed within a Bayesian framework using a Markov chain Monte Carlo (MCMC) algorithm to derive the posterior distributions for unknown parameters. For each model we used at least 4 million Markov chain iterations and assessed convergence by visual inspection of the trace plots.

If ***φ***_*j*_ represents the set of unknown parameters for model *j* and if *p*(***φ***_*j*_ |*D*) is the posterior distribution of these parameters given model *j*, data *D* and priors *p*(***φ***_*j*_) then

$$p(\varphi_{j}|D)\propto p(D|\varphi_{j})p(\varphi_{j})$$

For given parameter values, ***φ***_*j*_, a system of differential equations was used to determine the expected number of new infectious people in each seven-day period, *Z*_*i*_ (S1 Appendix).

For the likelihood term, *p*(*D|****φ***_*j*_), we assumed that the observed number of cases in each camp in week *i* followed a negative binomial distribution, with a mean given by the product of *Z*_*i*_ and *π*, the proportion of infections which are reported. In all cases parameters to be estimated included this proportion (*π*), the dispersion parameter of the negative binomial distribution, the time of the first case in each camp, the rates of leaving latently infected and infectious compartments, and a transmission parameter, *β*. Compared to the baseline model (Model 1), Models 2a and 2b estimated three additional parameter corresponding to the camp-specific estimates of the water and sanitation intervention effect on rates of transmission assuming stepwise effects associated with the intervention, and effects that scaled in proportion to the water sources per person respectively. This analysis was intended to evaluate the evidence that this intervention contributed to epidemic control.

Model 5 estimated one additional parameter (the proportion of transmission events that resulted from contacts with individuals in the first infectious period). Model 6 estimated two additional parameters: *λ*, the rate of increase in the contamination of the saturating environment per infected host and *υ*, the rate of loss of contamination from this environmental reservoir.

### Intervention analysis

To evaluate the potential impact of vaccination we used estimates of vaccine effectiveness after two and three doses derived from data in Zhu et al [23] (Table 2). This gave posterior means (and central 95% credible intervals) of 80.2% (16.4%, 99.6%) for two doses and 93.3% (74.3%, 99.8%) for three (S2 Appendix) and assumed 90% coverage for the first two doses in target groups. The intervals between the first and second and the second and third doses were one and five months respectively. There was no evidence of any effect of a single dose of vaccine in the clinical trial, so this was excluded from the analysis. In the absence of evidence to the contrary, we assumed vaccine effectiveness did not vary by age. We assumed no loss of vaccine or infection derived immunity over the timescales considered, in accordance with findings of long-term follow-up studies which found consistent vaccine-induced protection over 4.5 years and a slow rate of decline of immunity derived from infections [28,29].

**Table 2:** Assumptions used for evaluation of vaccination policies.

Assumption		Notes
Vaccine effectiveness (1 dose)	0	No data available
Vaccine effectiveness, 2 doses mean (95% CrI)	80.2% (16.4%, 99.6%)	Derived from Zhu et al. 2010 [23] (see S2 Appendix)
Vaccine effectiveness (3 doses)	93.3% (74.3%, 99.8%)	Derived from Zhu et al. 2010 [23] (see S2 Appendix)
Time from first to second dose	4 weeks	Zhu et al. 2010 [23]
Time from second to third dose (if given)	22 weeks	Zhu et al. 2010 [23]
Proportion of groups targeted to receive vaccine who get 1st two doses	90%	Assumption based on experience at other camps for displaced persons
Proportion of groups targeted to receive vaccine who get 3rd dose (if given)	90%	As above
Time from vaccination to resulting immunity (if effective)	Two weeks	Immunity assumed to be generated instantaneously two weeks after second dose and two weeks after third dose
Number of reported cases before reactive vaccination starts	50 or 100	Assumption
Percentage of population pregnant	3%	United Nations data^a^
Proportion of population aged ≥ 15 years	51%	United Nations data^a^
Proportion of population aged > 65 years	2%	United Nations data^a^
Probability of symptomatic illness given infection	0.20 (0.17, 0.23)	Rein et al 2010 [6]. In simulations represented by a Beta(133.76, 541.81) distribution which gives the same mean and 95% CrI.
Probability of death given symptomatic infection if not pregnant	0.019 (0.017, 0.021)	Rein et al 2010 [6]. In simulations represented by a Beta(340.79, 17595.66) distribution which gives the same mean and 95% CrI.
Probability of death given symptomatic infection if not pregnant	0.20 (0.17, 0.23)	Rein et al 2010 [6]. In simulations represented by a Beta(143.68, 581.99) distribution which gives the same mean and 95% CrI.

^a^ United Nations, Department of Economic and Social Affairs, Population Division (2015). World Population Prospects: The 2015 Revision, custom data acquired via website (https://esa.un.org/unpd/wpp/DataQuery)

**Fig 2:**
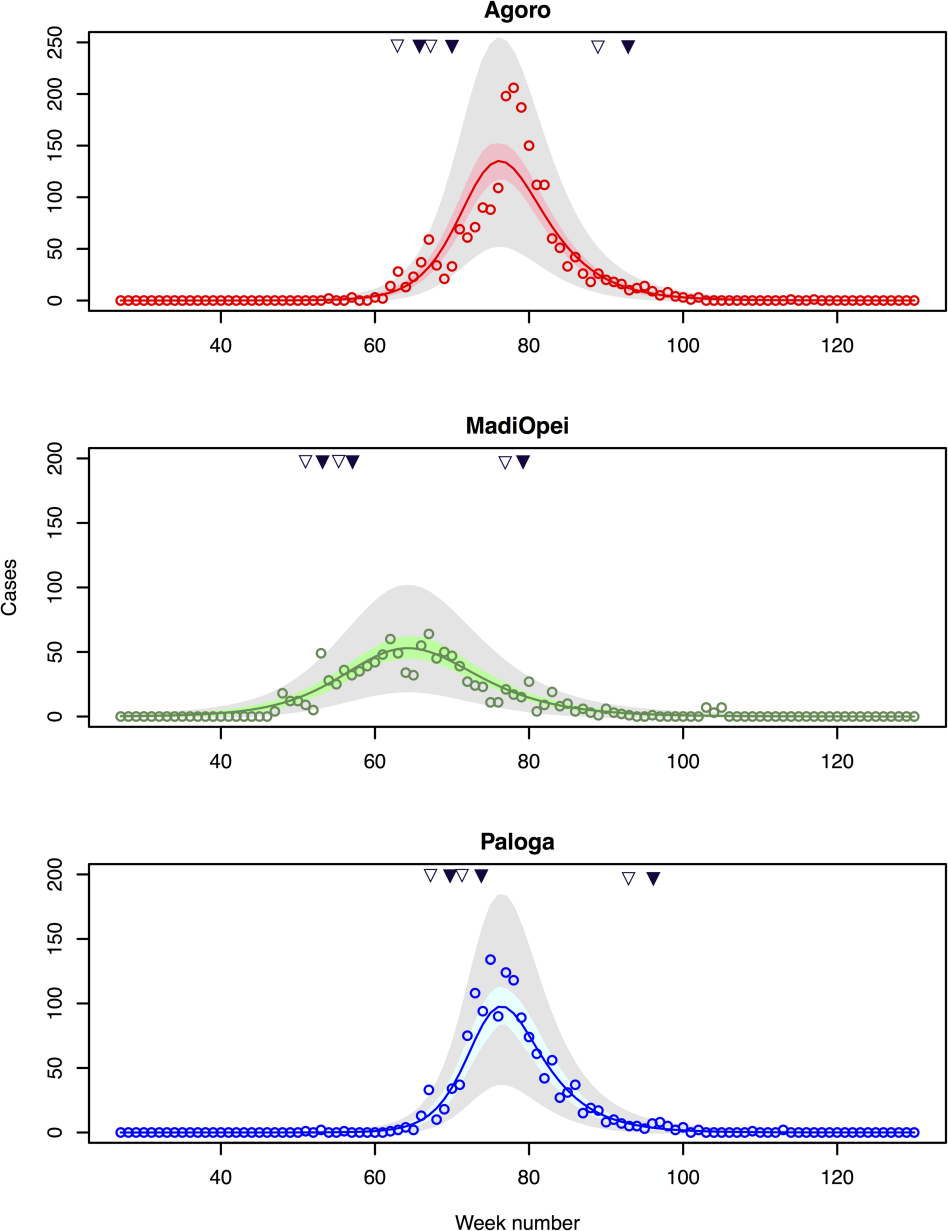
Observed and predicted hepatitis E cases. Observed weekly cases (circles) and expected weekly cases at the three sites based on the baseline transmission model (solid line) and 95% Credible Intervals for the mean (shaded coloured region) and for the predicted number of cases (shaded grey region). Week number 1 corresponds to the first week of 2007. Times when the first, second and third vaccine doses would have been given under reactive vaccination strategies triggered by 50 or 100 cumulative cases are shown by open and closed triangles respectively.

Case fatality ratios amongst those pregnant and those not pregnant were derived from the meta-analysis of Rein et al. [6]. We assumed a threshold of 50 or 100 reported cases as the starting point for reactive vaccination. Other assumptions are given in Table 2. Regardless of the threshold number of cases for initiating vaccination, a three dose vaccination strategy was considered incompatible with reactive vaccination because the third dose would have to be given close to the end of the epidemic (Fig 2). We therefore only consider two dose reactive vaccination scenarios. A web application that uses the baseline model and allows for the simulation of the effects of different vaccination strategies (including three dose reactive vaccination) under user-specified assumptions is available at https://moru.shinyapps.io/HEVmodel/.

In all simulations we accounted for uncertainty in model parameters by drawing these from the posterior distributions obtained by fitting the model to data from the three camps. Analysis was performed in R [30]. Model code is available at https://github.com/BenSCooper/HEVmodel.

## Results

**Fig 3:**
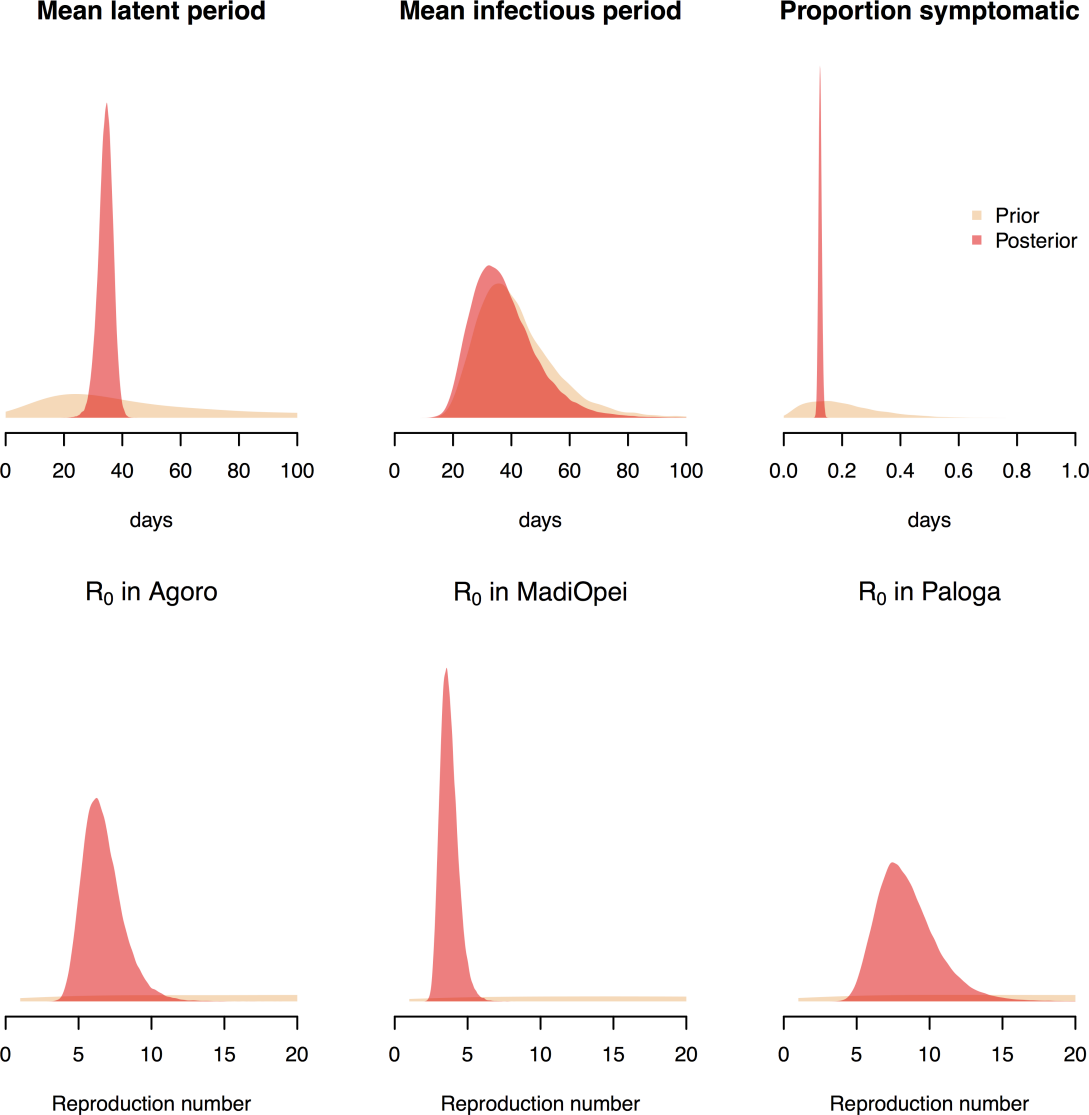
Prior and posterior distributions for key epidemiological parameters. Estimates are derived using the baseline SEIR model with informative priors: the mean latent period, the mean infectious period and the proportion symptomatic (top row) are assumed to share the same distributions at the three camps. Posterior distributions of the basic reproduction number (R_0_) are allowed to vary by camp (bottom row).

The baseline model (Model 1a) gave good fits to the three epidemic curves, with the observed number of weekly cases usually within the 95% prediction intervals for observed cases (grey shaded region) (Fig 2). Under the baseline model the mean latent period was estimated to be 34 days, 95% CrI (29, 39) (Fig 3 and Table 3; example MCMC output is shown in S3 Appendix). Similar estimates were obtained in sensitivity analyses, though when the prior for the infectious period was based on a single patient with HEV genotype 1 (with 22 days post-onset shedding) the posteriors for the infectious period and camp-specific reproduction numbers gave more support to lower values than under baseline assumptions (Model 1b, Table 3). The effect of assuming prior immunity in the population was to slightly increase estimates of the proportion of infections which are reported (from 13% with no immunity to 18% if 30% of the population were initially immune) and to lead to higher estimates of basic reproduction numbers. These were inflated by about 50% if 30% of the population were assumed initially immune (S1 Table). A shorter mean latent period (19 days (10, 34)) was inferred if alternative distributional assumptions were made about the latent period (Model 3, Table 3). The mean infectious period was estimated to be 36 days, 95% CrI (21, 64), in the baseline model, though this reduced to 27 days (21, 37) under different distributional assumptions (Model 4, Table 3). The estimated proportion of infections reported was 12.5% (11.4%, 13.6%) in the baseline model and similar in all sensitivity analyses. Under baseline assumptions the basic reproduction numbers were estimated to be similar in two of the three camps (Agoro 6.5 (4.5, 9.9); Paloga 8.5 (5.3, 11.4)), but smaller in Madi Opei (3.7 (2.8, 5.1)). Central estimates for these reproduction numbers were similar in most sensitivity analyses (Table 3), though higher in models that explicitly accounted for two modes of transmission (Table 4).

**Table 3:** Results for models 1–5 (no saturating environmental reservoir).

	Model 1a: SEIR Baseline model with prior for infectious period based on HEV shedding data from 11 patients [20])	Model 1b: SEIR Baseline model, but with prior for infectious based on HEV shedding from a single patient with genotype 1. [20]	Model 2a: Accounting for water and sanitation intervention (stepwise effect)	Model 2b: Accounting for water and sanitation intervention (using water sources per person covariate)	Model 3: Erlang-distributed latent period	Model 4: Erlang-distributed infectious periods	Model 5: Time varying transmissibility
Parameter	Posterior median (95% CrI)						
Mean latent period (days)	34.4 (28.8, 38.8)	31.7 (24.7, 37.8)	36.3 (31.8, 40.2)	34.3 (28.6, 38.9)	19.1 (10.4, 34.2)	34.1 (30.8, 37.3)	31.3 (11.8, 37.0)
Mean infectious period (days)	35.9 (20.9, 64.4)	16.3 (4.3, 62.8)	31.5 (20.0, 52.5)	35.7 (21.8,61.5)	33.6 (28.4, 38.0)	27.3 (20.9, 36.6)	31.4 (22.3, 49.6)
Infections reported (%)	12.5 (11.4, 13.6)	12.9 (11.7, 14.4)	12.4 (11.4, 13.5)	12.3 (11.3, 13.5)	12.4 (11.3,13.7)	12.3 (11.3, 13.5)	12.3 (11.2, 13.5)
R_0_ Agoro R_0_ Madi Opei R_0_ Paloga	6.5 (4.5, 9.9) 3.7 (2.8, 5.1) 8.5 (5.3,11.4)	4.3 (2.8, 9.9) 2.6 (2.0, 5.2) 5.0 (3.1, 13.5)	5.5 (4.1, 8.3) 4.1 (3.2, 5.2) 7.6 (5.6,11.1)	6.7 (4.6, 9.7) 3.5 (2.6, 5.3) 7.8 (5.0, 12.8)	5.3 (3.6, 8.6) 3.1 (2.4, 4.1) 6.6 (4.1, 12.1)	5.9 (4.5, 7.9) 3.2 (2.7, 4.0) 7.0 (5.2, 9.5)	5.8 (3.8, 9.3) 3.2 (2.4, 4.2) 7.3 (4.3, 12.7)
Day 1st infection Agoro Day 1st infection Madi Opei Day 1st infection Paloga	278 (257, 298) 62 (16, 103) 327 (305, 343)	288 (262, 309) 80 (24, 124) 332 (310, 343)	263 (225, 231) 106 (61,145) 305 (323, 330)	279 (258, 298) 87 (44, 123) 322 (302, 330)	270 (245, 292) 68 (16, 113) 315 (292, 329)	281 (268, 293) 78 (34, 114) 318 (299, 329)	271 (246, 292) 64 (13, 109) 315 (292, 329)
Dispersion parameter	7.3 (4.9, 11.0)	7.3 (4.9, 10.9)	7.0 (4.6, 10.6)	6.7 (4.5, 10.0)	6.4 (4.1, 9.9)	6.3 (4.2, 9.3)	6.3 (4.2, 9.4)
WATSAN effect Agoro^1^	-	-	1.5 (0.9,2.5)	1.00 (0.82,1.22)	-	-	-
WATSAN effect Madi Opei^1^	-	-	2.7 (0.6, 11.5)	1.07 (0.89, 1.27)	-	-	-
WATSAN effect Paloga^1^	-	-	0.9 (0.001, 2063)	0.99 (0.81, 1.20)	-	-	-
Proportion of transmission in 1st infectious period	-	-	-	-	-	-	0.56 (0.04, 0.98)
Number of parameters estimated	10	10	13	13	10	10	11
DIC^2^	1140	1140	1163	1160	1165	1145	1167

^1^ In model 2a the WATSAN effect reported is the estimate of ratio of the per capita transmission rate in post-WATSAN intervention period compared to that in the pre-intervention period (so values less than one indicate reduced transmission post WATSAN intervention). In model 2b the estimated effect represents the rate ratio associated with a one-unit increase in the estimated number of drinking water sources per 1000 people. In both models R_0_ values reported assume no effect of the WATSAN intervention.

^2^ Deviance Information Criterion (lower values indicate better fit). Here the DIC is calculated as $\bar{D}+p_{v}$, where $\bar{D}$ is the mean posterior deviance and *p*_*v*_ is var(*D*/2) which is an estimate of the effective number of parameters (Gelman et al 2004 [31]).

**Table 4:** Selected results for model 6 (saturating environmental reservoir).

	Model 6: SEIRW	Model 6: SEIRW	Model 6: SEIRW	Model 6: SEIRW	Model 6: SEIRW
Prior for *𝜐*, rate of loss of contamination from environmental reservoir^1^. (mean duration of persistence in environmental reservoir)	w3 (14 days)	w3 (14 days)	w3 (14 days)	w4 (28 days)	w5 (56 days)
Prior for initial proportion of transmission that occurs via contamination of the saturating environmental reservoir^1^	p3 (mean 50%)	p4 (mean 75%)	p5 (mean 95%)	p5 (mean 95%)	p5 (mean 95%)
Parameter	Posterior median (95% CrI)				
Mean latent period (days)	31.2 (22.2, 37.3)	31.9 (24.0, 37.6)	32.8 (20.8, 38.7)	33.0 (22.8, 38.7)	33.2 (24.9, 38.6)
Mean infectious period (days)	36.2 (22.8, 62.3)	44.5 (25.3, 79.9)	36.2 (21.0, 68.4)	35.5(21.2, 68.7)	38.5 (21.2, 84.1)
Infections reported (%)	12.2 (11.2, 13.4)	12.1 (11.1, 13.2)	12.2 (11.2, 13.4)	12.2 (11.1, 13.4)	12.2 (11.1, 13.3)
R_0_ direct camp 1 R_0_ direct camp 2 R_0_ direct camp 3	5.3 (3.5, 8.3) 3.2 (2.1, 4.8) 5.8 (3.6, 9.5)	5.3 (2.1, 8.8) 3.0 (1.0, 4.9) 5.7 (2.1, 10.0)	0.8 (0.3, 2.4) 0.4 (0.1, 1.1) 0.9 (0.3, 2.4)	1.0 (0.3, 2.6) 0.5 (0.2, 1.1) 1.0 (0.4, 2.6)	1.7 (0.6, 6.9) 0.8 (0.3, 3.7) 1.7 (0.6, 7.4)
R_0_ indirect camp 1 R_0_ indirect camp 2 R_0_ indirect camp 3	4.9 (2.4, 10.3) 3.7 (1.9, 7.4) 4.5 (2.3, 8.7)	11.2 (4.8, 29.6) 9.6 (3.2, 31.1) 9.5 (4.8, 19.7)	12.8 (7.1, 30.3) 6.3 (3.8, 14.6) 13.9 (7.4, 32.5)	16.2 (9.9, 29.7) 7.7 (4.9, 14.4) 17.7 (10.5, 31.2)	25.9 (15.1, 74.2) 12.2 (7.0, 76.4) 28.1 (16.0, 58.9)
Mean duration virus remains viable in environment (days)	15.7 (7.9, 35.7)	19.5 (9.0, 46.6)	17.9 (8.3, 47.4)	29.2 (22.3, 39.4)	58.8 (44.6, 78.4)
Rate of contamination of saturating environment per infected hosts	0.18 (0.02, 0.90)	0.08 (0.005, 0.89)	0.01 (0.001, 0.02)	0.01 (0.002, 0.02)	0.01 (0.002, 0.15)
Day 1st infection Agoro Day 1st infection Madi Opei Day 1st infection Paloga	302 (280, 321) 155 (105, 196) 326 (312, 330)	308 (279, 333) 177 (88, 233) 326 (311, 330)	294 (264, 317) 111 (45, 162) 320 (302, 330)	295 (265, 316) 117 (44, 163) 322 (302, 329)	295 (268, 321) 124 (56, 209) 322 (301, 330)
Dispersion parameter	6.7 (4.5, 9.9)	6.5 (4.4, 9.7)	6.3 (4.2, 9.4)	6.3 (4.2, 9.2)	6.3 (4.2, 9.3)
DIC^2^	1162	1167	1160	1160	1162

^1^See Table 1 for definitions of priors.

^2^ Deviance Information Criterion (lower values indicate better fit). See Table 3 footnote for details.

Analysis of the data using models explicitly accounting for the water and sanitation intervention (Models 2a and 2b, Table 3) did not provide evidence that these interventions were effective in reducing transmission. However, results were unable to rule out both substantial beneficial and harmful effects (WATSAN coefficients less than one and greater than one respectively), indicating that the data contained little information about the effects of the water and sanitation responses on transmission. This reflects the fact that in all three camps the interventions were not fully in place until the HEV epidemics were almost over (S1 Fig). Fitting the models accounting for a second mode of transmission corresponding to an unobserved environmental reservoir showed that the data were only consistent with the majority of transmission occurring via this route if mean persistence of viable virus in this environmental reservoir was two weeks or more (S2 Fig).

**Fig 4:**
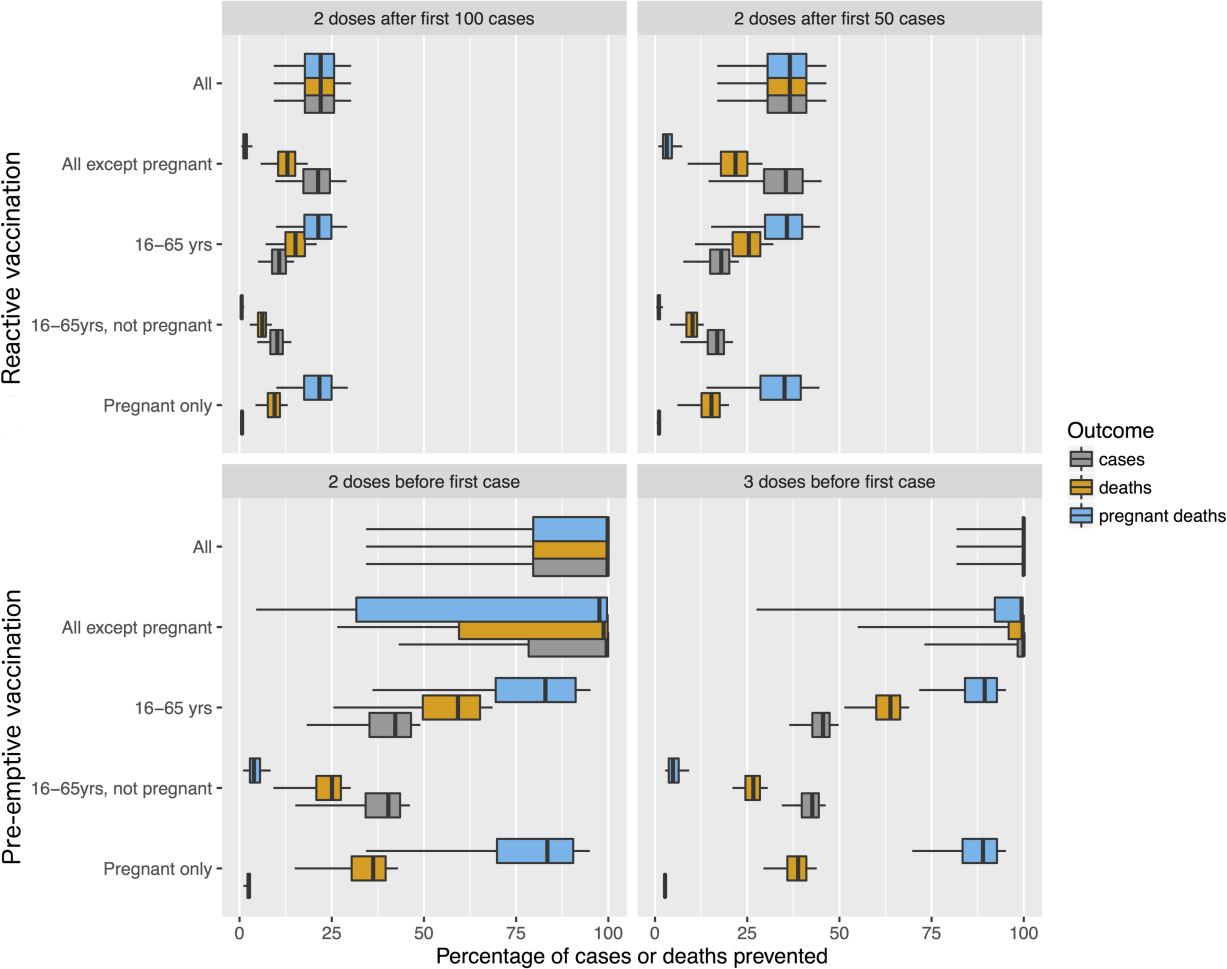
Percentage of hepatitis E cases, deaths and deaths of pregnant women prevented by different vaccination strategies. Estimates are derived using posterior distributions of model parameters obtained by fitting the baseline model with weakly informative priors to data from Agoro. Boxplots show the median (central bar), interquartile range (extent of coloured box) and 5th and 95th percentiles (whiskers).

Considering the potential effects of different vaccine usage scenarios, under the baseline model, reactive vaccination (assuming two doses after the first 100 or 50 cases) was capable of producing important though relatively modest reductions in cases and deaths (Fig 4, top row). These reductions were sensitive to the threshold number of cases before reactive vaccination was initiated: when the threshold was 100, reductions in mortality compared to no vaccination scenarios were unlikely to exceed 25% even assuming the vaccine was given without age or pregnancy restrictions; for a threshold of 50, mortality reductions of about 40% were plausible. In contrast, with no age-restriction on vaccine recipients and pre-emptive vaccination (Fig 4, bottom row), reductions in mortality of 100% were possible indicating that the herd immunity threshold had been reached. This was true whether two or three doses were administered, though in the former case uncertainty was far larger reflecting the lower precision in the estimated vaccine effectiveness of two doses (S2 Appendix).

The impact of excluding pregnant women from the population to vaccinate varied with scenario. In cases where vaccination had a high chance of achieving herd immunity (i.e. when vaccine was used pre-emptively without age restriction) excluding pregnant women had only a moderate negative impact on reductions in mortality. In contrast, when herd immunity was less likely to be obtained through vaccination (i.e. when the vaccine was used reactively or with age restrictions) excluding pregnant women led to substantially smaller reductions in total mortality (and much smaller reductions in mortality in pregnant women).

Comparing vaccination policies with and without age restrictions, restricting receipt of the vaccine to those between the ages of 16 and 65 years had a small impact on reductions in mortality in the reactive vaccination strategy, but a far larger negative impact on the pre-emptive vaccination strategy, a consequence of reducing the chance of achieving herd immunity in these latter strategies.

These broad conclusions about the impact of different vaccination strategies were robust to precise details of model specification. In particular, under all 25 scenarios in the model with two modes of transmission (Model 6), similar patterns were seen (S3 Fig), though the smallest reductions in mortality were seen when priors expressed the belief that most transmission was via this environmental route and viral persistence in this environment was long.

## Discussion

Our results indicate that in displaced population settings HEV can be highly transmissible. In one camp (Paloga) the estimated mean number of secondary cases per primary case at the start of the epidemic (the basic reproduction number, R_0_,) exceeded 6.5 under all model assumptions. This number has important implications for vaccination policies. To achieve herd immunity requires successful immunization of a percentage of individuals given by 100-100/ R_0_ [32]. Thus, even taking the optimistic R_0_ value of 6.5, we would require 85% of the population to be effectively immunized to achieve herd immunity. Herd immunity is desirable as it means a major epidemic will not be possible even though many in the population remain susceptible to infection. It is of particular relevance here because HEV-infected pregnant women face a greatly increased risk of mortality but the safety and efficacy of the vaccine in pregnant women remains to be established.

Even when restricting vaccine use to non-pregnant 16–65 year olds (for which the vaccine is licenced in China), the benefits could be substantial, with reductions in mortality of over 40% likely under baseline assumptions given pre-emptive vaccination with three doses. A robust finding was that pre-emptive vaccination was much more effective at preventing HEV cases and deaths than reactive vaccination. Estimates of reductions in mortality were based on the case fatality estimates taken from the meta-analysis of HEV genotypes 1 and 2 outbreaks by Rein et al [6], as we lacked reliable local mortality data. In practice mortality rates might be expected to vary considerably between locations, and substantially higher mortality rates have been reported by some studies [7,33]. Evaluation of the potential benefits of HEV vaccination in an emergency setting should take into account the best estimates of local mortality rates.

Our work sheds light on other important aspects of HEV epidemiology: we found consistent evidence that the mean latent period is between about 20 and 40 days and that a little over 10% of individuals infected with HEV are identified as cases. The data were less informative about the mean infectious period, though were consistent with the range 20–70 days suggested by previous data.

Previous epidemiological investigations had suggested that household-level person-to-person spread may have been important in this epidemic [21,22]. Our results show that the epidemic curves in all three camps can be reproduced without positing the existence of a saturating environmental reservoir, and models with such a reservoir did not improve fits to data. In fact, models 1 and 4 had substantially lower DICs than more complex models (including those explicitly accounting for an environmental reservoir) suggesting that these simpler models should be preferred in the absence of strong evidence that a saturating environmental reservoir played a role in this epidemic. We found no evidence that the water and sanitation interventions reduced transmission, though these interventions were introduced late and the data provide little evidence for or against their effectiveness. Previous modelling has shown that such interventions have the potential to be highly effective if assumed to be capable of interrupting the dominant mode of transmission [34]. More recently, a matched case control study of HEV infection in a 2014 outbreak in a predominantly rural-nomadic population in Napak District, Uganda found that eating roadside food, drinking untreated water, and not always cleaning utensils were strongly associated with risk of HEV infection [33]. These findings are consistent with fecal oral transmission being the dominant mode of spread, and suggest that both contaminated drinking water and household transmission might play important roles. While our analysis does not allow us to quantify the relative importance of different transmission routes, the results do suggest that if an environmental reservoir was important for transmission in this epidemic the current number of infectious people was a good proxy for the infection pressure from the environment.

Given the uncertainties about transmission routes and fundamental methodological challenges in making inferences about an unobserved environmental reservoir [35], we performed extensive sensitivity analyses. While this did not make it possible to quantify the relative importance of different transmission routes it did shed light on the circumstances where a saturating reservoir would be consistent with the observed epidemic data. A key finding is that if the mean persistence of viable virus in the environment was seven days or fewer, the saturating environmental reservoir was estimated to play only a minor role in the epidemic. If mean persistence was two weeks or more, however, the data were compatible with such an environmental reservoir representing the dominant mode of transmission.

Strengths of our work include the use of high quality epidemic data, extensive sensitivity analysis, and an analysis that allows us to incorporate information from previous HEV investigations. This work also has important limitations. First, estimates of HEV vaccine effectiveness from a trial in China may not generalise to a typical emergency setting or to age/ethnic groups not included in the trial. Moreover, the model assumes that when an individual is vaccinated they have an all-or-nothing response: either full protection against disease and ability to transmit infection or no protection. In practice, components of vaccine protection may be more complex [36], and it is not known whether vaccinated individuals developing subclinical infections have reduced transmissibility [28]. Second, our analysis neglects age, spatial and household structuring, behavioural, biological and temporal heterogeneities that might affect HEV transmission. These may all play important roles in HEV epidemiology in emergency settings but we lacked data of sufficient resolution to meaningfully incorporate them. Delineating such factors is an important area for future work. There is some evidence that disease severity is affected by age [10]. By analogy with other viral infections, we might anticipate that transmissibility, infectious period and vaccine effectiveness also vary be age. Such differences could have an important influence on the relative effectiveness of different vaccination policies. Understanding such age effects, and incorporating them into models should be considered a priority. We assumed no prior immunity though lacked pre-epidemic sera to enable us to assess this assumption. If some people were immune prior to the epidemic, we are likely to have underestimated the basic reproduction numbers. Third, lacking any data on the efficacy of a single vaccine dose, we assumed it conferred no protective effect. This is a conservative assumption and may mean that we have underestimated the potential vaccine benefits. Given the logistical challenges of delivering three doses over a six month period, future research to better quantify the value of one and two doses of vaccine and perhaps a reduced interval between doses 2 and 3 would be valuable. Finally, it is not clear how relevant the modelling framework used here will be for community outbreaks where HEV is already endemic, such as the recent urban outbreak in Chad [37]; further work is needed to evaluate the potential impact of hepatitis E vaccination in different contexts.

In conclusion, this work has shown a high transmission potential for HEV in displaced population settings, shed light on important natural history parameters, and has shown that mass vaccination campaigns in such high risk populations have the potential to lead to substantial reductions in mortality.

In 2015 the Strategic Advisory Group of Experts on immunization could not recommend the routine use of the vaccine for population sub-groups including children aged less than 16 years and pregnant women but emphasized that the use of the vaccine during outbreaks of hepatitis E should be considered [38]. Our findings show that such an intervention, while not as effective as pre-emptive vaccination, could nonetheless have a major impact, particularly if vaccination can be safely extended to high risk groups excluded from vaccine trials. In particular, these results underline the need to prioritise evaluations of the vaccine in pregnant women [39].

## Supporting information

S1 Fig Water and sanitation data. The figure shows the number of water sources (taps) per person in each camp and the timing of the water and sanitation intervention relative to the epidemic curves. The top row shows the water sources per person from the three camps by week number (from January 2007). Absence of a black line segment indicates lack of data; the red dashed line corresponds to minimal Sphere recommendations of no more than 250 people per tap assuming a flow of 7.5 litres per minute (http://www.spherehandbook.org/en/water-supply-standard-1-access-and-water-quantity/). The broken green line shows the start time of the water and sanitation intervention in the three camps. The bottom row shows the number of Hepatitis E cases reported in the three camps over the same period. For periods prior to the collection of data we assumed that the number of water sources per person was constant and equal to the first observed value in the same camp. Similarly, for periods after collection of water and sanitation data, we assumed that the number of water sources per person was equal to the most recent recorded value in each camp.
(TIFF)

S2 Fig Prior and posterior distributions for the percentage of transmission occurring by exposure to a saturating environmental compartment under model 6 (SEIRW). Priors p1 to p5 correspond to varying the initial percentage of transmission occurring via this environmental route between mean values of 5% and 95% when the population is fully susceptible. Priors w1 to w5 correspond to varying the persistence of viable virus in this environmental reservoir, with mean durations ranging from two days to eight weeks. Prior and posterior distributions shown correspond to the proportion of transmission that occurs via this environmental route in completed epidemics at Agoro (note that the priors shown differ from the priors of the initial percentage of transmission via the environmental, because saturation of the environmental reservoir will tend to decrease the relative importance of this route per case over time).
(TIFF)

S3 Fig Percentage of hepatitis E cases, deaths and deaths of pregnant women prevented by different pre-emptive vaccination strategies under model 6 (SEIRW model). (TIFF)

S1 Appendix Model equations. (PDF)

S2 Appendix Vaccine effectiveness assumptions. (PDF)

S3 Appendix MCMC samples. (PDF)

S1 Table Sensitivity analysis assuming prior immunity in the population. (PDF)

## References

[1] Connolly MAA, Gayer M, Ryan MJ, Salama P, Spiegel P, Heymann DL. Communicable diseases in complex emergencies: impact and challenges. Lancet. 2004; 364: 1974–1983. 10.1016/S0140-6736(04)17481-3 15567014

[2] Basnyat B, Dalton HR, Kamar N, Rein DB, Labrique A, Farrar J, et al. Nepali earthquakes and the risk of an epidemic of hepatitis E. Lancet. 2015; 385: 2572–2573. 10.1016/S0140-6736(15)61110-2 26091742

[3] Scobie L, Dalton HR. Hepatitis E: source and route of infection, clinical manifestations and new developments. J Viral Hepat. 2013;20: 1–11. 10.1111/jvh.12024 23231079

[4] Balayan MS. Epidemiology of hepatitis E virus infection. J Viral Hepat. 1997;4: 155–165. 9181524 10.1046/j.1365-2893.1997.00145.x

[5] Labrique AB, Sikder SS, Krain LJ, West KP Jr, Christian P, Rashid M, et al. Hepatitis E, a vaccine-preventable cause of maternal deaths. Emerg Infect Diseases. 2012;18: 1401–1404. 22931753 10.3201/eid1809.120241 PMC3437697

[6] Rein D, Stevens G, Theaker J, Wittenborn J, Wiersma S. The global burden of hepatitis E virus genotypes 1 and 2 in 2005. Hepatology. 2012;55: 988–997. 10.1002/hep.25505 22121109

[7] Boccia D, Guthmann JP, Klovstad H, Hamid N, Tatay M, Ciglenecki I, et al. High mortality associated with an outbreak of hepatitis E among displaced persons in Darfur, Sudan. Clin Infect Dis. 2006; 42: 1679–1684. 10.1086/504322 16705571

[8] Guthmann JP, Klovstad H, Boccia D, Hamid N, Pinoges L, Nizou JY, et al. A large outbreak of hepatitis E among a displaced population in Darfur, Sudan, 2004: the role of water treatment methods. Clin Infect Dis. 2006;42: 1685–1691. 10.1086/504321 16705572

[9] CDC. Investigation of hepatitis E outbreak among refugees—Upper Nile, South Sudan, 2012–2013. MMWR Morb Mortal Wkly Rep. 2013;62: 581–586. 23884344 PMC4604969

[10] Teshale EH, Howard CM, Grytdal SP, Handzel TR, Barry V, Kamili S, et al. Hepatitis E epidemic, Uganda. Emerg Infect Dis. 2010;16: 126–129. 10.3201/eid1601.090764 20031058 PMC2874362

[11] Jin H, Zhao Y, Zhang X, Wang B, Liu P. Case-fatality risk of pregnant women with acute viral hepatitis type E: a systematic review and meta-analysis. Epidemiol Infect. 2016; 144: 2098–2106. 10.1017/S0950268816000418 26939626 PMC9150575

[12] UNHCR. http://www.unhcr.org/uk/figures-at-a-glance.html (date accessed: 26/1/2017).

[13] Balayan MS, Andjaparidze AG, Savinskaya SS, Ketiladze ES, Braginsky DM, Savinov AP, et al. Evidence for a virus in non-A, non-B hepatitis transmitted via the fecal-oral route. Intervirology. 1983;20: 23–31. 10.1159/000149370 6409836

[14] Chauhan A, Jameel S, Dilawari JB, Chawla YK, Kaur U, Ganguly NK. Hepatitis E virus transmission to a volunteer. Lancet. 1993;341: 149–150. 8093748 10.1016/0140-6736(93)90008-5

[15] Nanda SK, Ansari IH, Acharya SK, Jameel S, Panda SK. Protracted viremia during acute sporadic hepatitis E virus infection. Gastroenterology. 1995;108: 225–230. 7806046 10.1016/0016-5085(95)90028-4

[16] Aggarwal R, Kini D, Sofat S, Naik SR, Krawczynski K. Duration of viraemia and faecal viral excretion in acute hepatitis E. Lancet. 2000;356: 1081–1082. 10.1016/S0140-6736(00)02737-9 11009149

[17] Clayson ET, Myint KS, Snitbhan R, Vaughn DW, Innis BL, Chan L, et al. Viremia, fecal shedding, and IgM and IgG responses in patients with hepatitis E. J Infect Dis. 1995;172: 927–933. 7561211 10.1093/infdis/172.4.927

[18] Chandra N, Sharma A, Malhotra B, Rai R. Dynamics of HEV viremia, fecal shedding and its relationship with transaminases and antibody response in patients with sporadic acute hepatitis E. Virol J. 2010;7: 213 10.1186/1743-422X-7-213 20815928 PMC2940811

[19] Aggarwal R, Krawczynski K. Hepatitis E: An overview and recent advances in clinical and laboratory research. J Gastroenterol Hepatol. 2000;15: 9–20. 10719741 10.1046/j.1440-1746.2000.02006.x

[20] Takahashi M, Tanaka T, Azuma M, Kusano E, Aikawa T, Shibayama T, et al. Prolonged fecal shedding of hepatitis E virus (HEV) during sporadic acute hepatitis E: evaluation of infectivity of HEV in fecal specimens in a cell culture system. J Clin Microbiol. 2007; 45: 3671–3679. 10.1128/JCM.01086-07 17728471 PMC2168470

[21] Howard CM, Handzel T, Hill VR, Grytdal SP, Blanton C, Kamili S, et al. Novel risk factors associated with hepatitis E virus infection in a large outbreak in northern Uganda: results from a case-control study and environmental analysis. Am J Trop Med Hyg. 2010;83: 1170–1173. 10.4269/ajtmh.2010.10-0384 21036857 PMC2963989

[22] Teshale EH, Grytdal SP, Howard C, Barry V, Kamili S, Drobeniuc J, et al Evidence of person-to-person transmission of hepatitis E virus during a large outbreak in Northern Uganda. Clin Infect Dis. 2010; 50: 1006–1010. 10.1086/651077 20178415

[23] Zhu FC, Zhang J, Zhang XF, Zhou C, Wang ZZ, Huang SJ, et al. Efficacy and safety of a recombinant hepatitis E vaccine in healthy adults: a large-scale, randomised, double-blind placebo-controlled, phase 3 trial. Lancet. 2010; 376: 895–902. 10.1016/S0140-6736(10)61030-6 20728932

[24] Wu T, Zhu FC, Huang SJ, Zhang XF, Wang ZZ, Zhang J, et al. Safety of the hepatitis E vaccine for pregnant women: a preliminary analysis. Hepatology. 2012;55: 2038 10.1002/hep.25522 22161542

[25] Nicand E, Grandadam M, Rey JL, Buisson Y. Viraemia and faecal shedding of HEV in symptom-free carriers. Lancet 2001;357: 68–69. 10.1016/S0140-6736(05)71568-3 11197383

[26] Teshale E, Grytdal S, Howard C, Handzel T, Kamili S. Hepatitis E outbreak investigation in Kitgum District, Northern Uganda. Report from Centers for Disease Control and Prevention; 2009.

[27] Parashar D, Khalkar P, Arankalle VA. Survival of hepatitis A and E viruses in soil samples. Clin Microbiol Infect. 2011;17: E1–4. 10.1111/j.1469-0691.2011.03652.x 21939469

[28] Zhang J, Zhang XF, Zhou C, Wang ZZ, Huang SJ, Yao X et al Protection against hepatitis E virus infection by naturally acquired and vaccine‐induced immunity. Clin Microbiol Infect. 2014; 20(6). 10.1111/1469-0691.12419 24118636

[29] Zhang J, Zhang XF, Huang SJ, Wu T, Hu YM, Wang ZZ, et al (2015). Long-term efficacy of a hepatitis E vaccine. New Engl J Med. 2015;372, 914–922. 10.1056/NEJMoa1406011 25738667

[30] R Core Team. R: A language and environment for statistical computing. R Foundation for Statistical Computing, Vienna, Austria 2015. URL URL https://www.R-project.org/. https://www.R-project.org/.

[31] Gelman A, Carlin JB, Stern HS, Rubin DB. Bayesian Data Analysis. Boca Raton: Chapman & Hall/CRC; 2004.

[32] Anderson RM, May RM. Infectious Diseases of Humans: Dynamics and Control. Oxford: Oxford University Press; 1992.

[33] Amanya G, Kizito S, Nabukenya I, Kalyango J, Atuheire C, Nansumba H, et al Risk factors, person, place and time characteristics associated with Hepatitis E Virus outbreak in Napak District, Uganda. BMC Infect Dis. 2017; 17: 451. 10.1186/s12879-017-2542-2 28651629 PMC5485539

[34] Nannyonga B, Sumpter DJ, Mugisha JY, Luboobi LS. The dynamics, causes and possible prevention of hepatitis E outbreaks. PLoS ONE. 2012;7:e41135 10.1371/journal.pone.0041135 22911752 PMC3404073

[35] Grad Y, Miller J, Lipsitch M. Cholera modeling: challenges to quantitative analysis and predicting the impact of interventions. Epidemiology. 2012;23: 523 10.1097/EDE.0b013e3182572581 22659546 PMC3380087

[36] Halloran ME, Struchiner CJ, Longini IM Jr. Study designs for evaluating different efficacy and effectiveness aspects of vaccines. Am J Epidemiol. 1997; 146: 789–803. 9384199 10.1093/oxfordjournals.aje.a009196

[37] Vernier L, Lenglet A, Hogema BM, Moussa AM, Ariti C, Vollmer S, et al Seroprevalence and risk factors of recent infection with hepatitis E virus during an acute outbreak in an urban setting in Chad, 2017. BMC Infect Dis. 2018; 18: 287 10.1186/s12879-018-3194-6 29940939 PMC6020170

[38] World Health Organization. Hepatitis E vaccine: WHO position paper, May 2015 Weekly epidemiological record 2015; 90,185–200.

[39] Nelson KE, Shih JW, Zhang J, Zhao Q, Xia N, Ticehurst JR, et al. Hepatitis E vaccine to prevent morbidity and mortality during epidemics. Open Forum Infect Dis. 2014;1: ofu098 10.1093/ofid/ofu098 25734166 PMC4324216

