# Supplementary figures and images for: Reactive and pre-emptive vaccination strategies to control hepatitis E infection in emergency and refugee settings: A modelling study
Source: PLoS Negl Trop Dis. 2018 Sep 25;12(9):e0006807. doi: 10.1371/journal.pntd.0006807 (PMC6173446; doi:10.1371/journal.pntd.0006807)

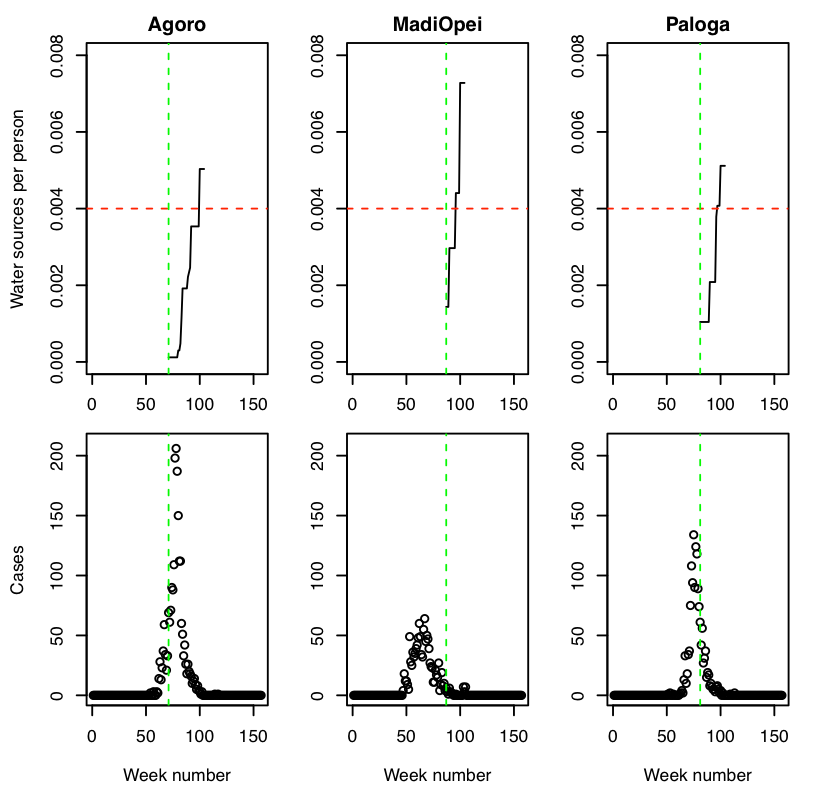

Supplement: S1 Fig — The figure shows the number of water sources (taps) per person in each camp and the timing of the water and sanitation intervention relative to the epidemic curves. The top row shows the water sources per person from the three camps by week number (from January 2007). Absence of a black line segment indicates lack of data; the red dashed line corresponds to minimal Sphere recommendations of no more than 250 people per tap assuming a flow of 7.5 litres per minute (http://www.spherehandbook.org/en/water-supply-standard-1-access-and-water-quantity/). The broken green line shows the start time of the water and sanitation intervention in the three camps. The bottom row shows the number of Hepatitis E cases reported in the three camps over the same period. For periods prior to the collection of data we assumed that the number of water sources per person was constant and equal to the first observed value in the same camp. Similarly, for periods after collection of water and sanitation data, we assumed that the number of water sources per person was equal to the most recent recorded value in each camp. (TIFF) [file pntd.0006807.s001.tiff]

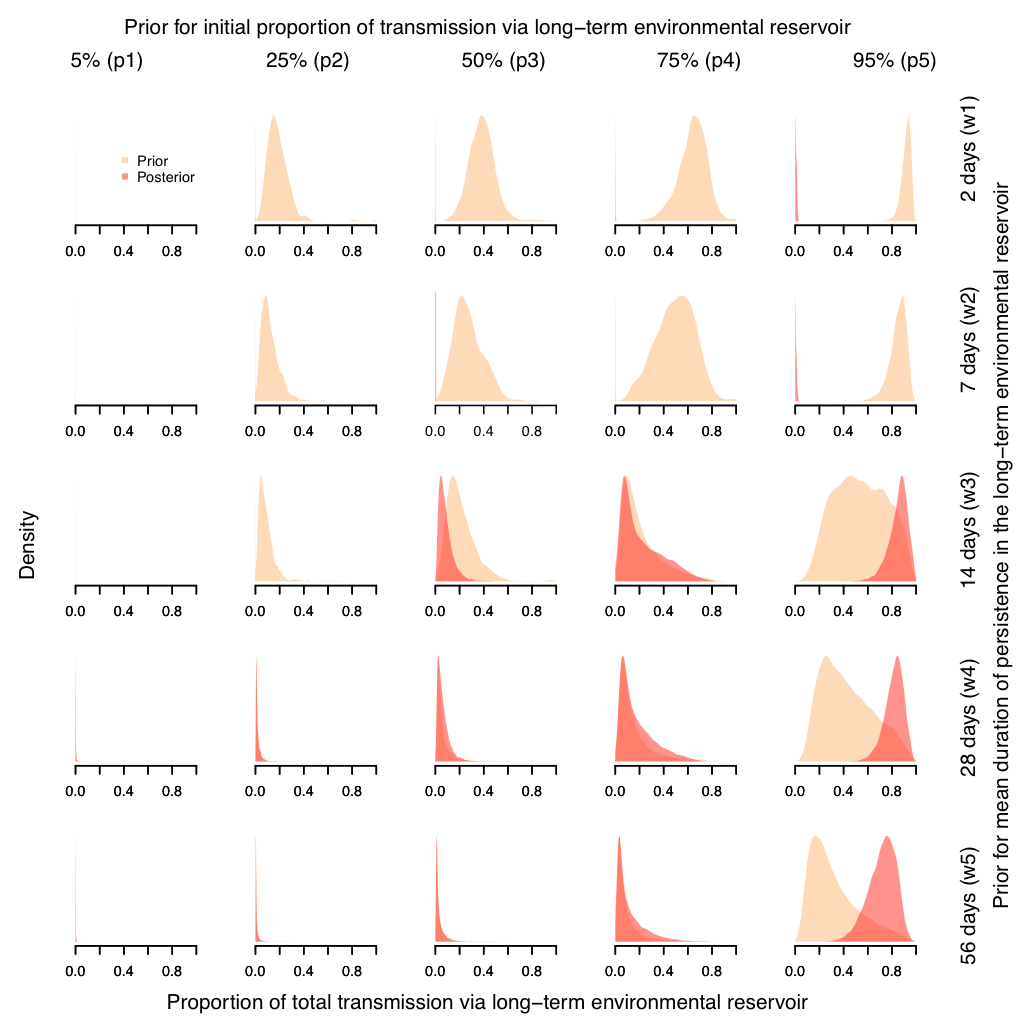

Supplement: S2 Fig — Priors p1 to p5 correspond to varying the initial percentage of transmission occurring via this environmental route between mean values of 5% and 95% when the population is fully susceptible. Priors w1 to w5 correspond to varying the persistence of viable virus in this environmental reservoir, with mean durations ranging from two days to eight weeks. Prior and posterior distributions shown correspond to the proportion of transmission that occurs via this environmental route in completed epidemics at Agoro (note that the priors shown differ from the priors of the initial percentage of transmission via the environmental, because saturation of the environmental reservoir will tend to decrease the relative importance of this route per case over time). (TIFF) [file pntd.0006807.s002.tiff]

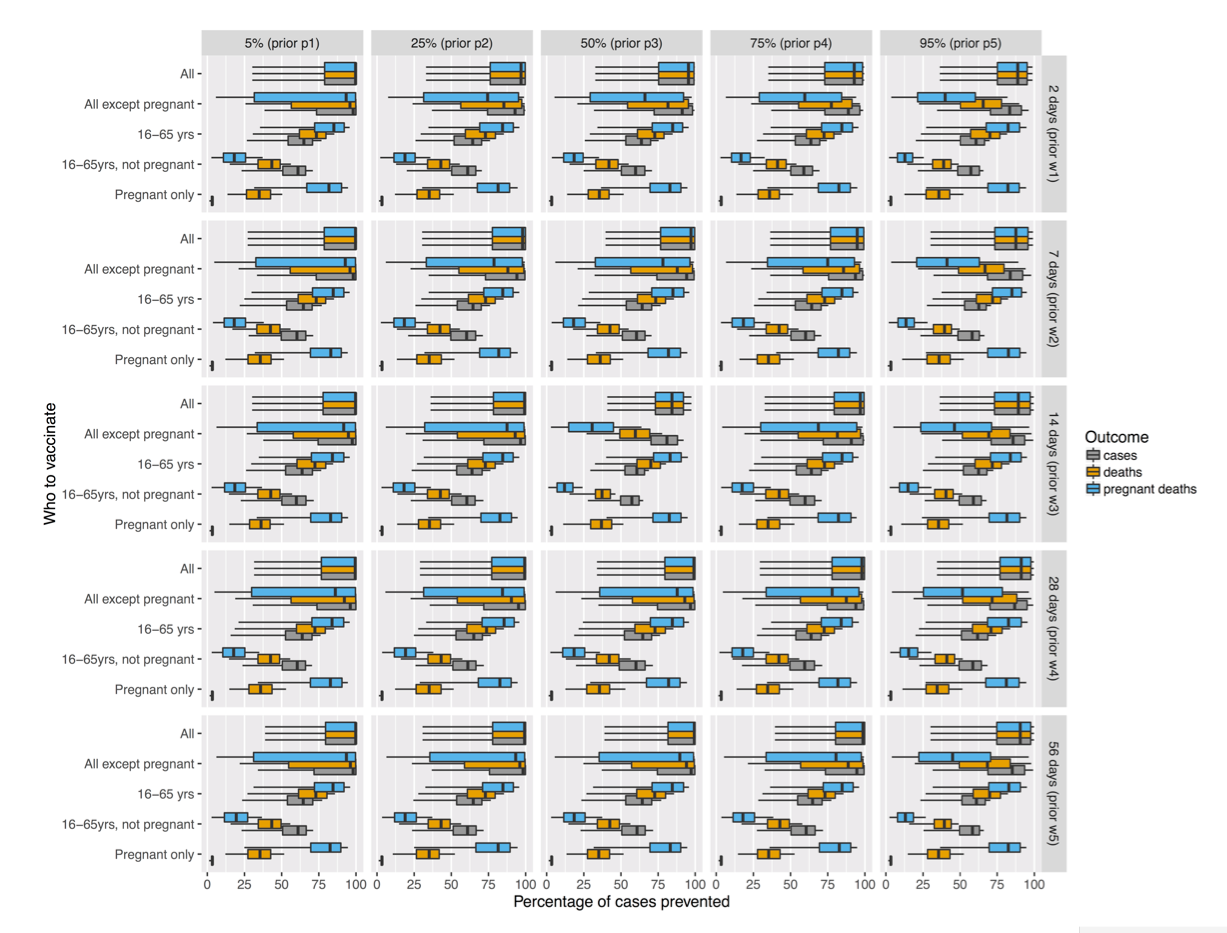

Supplement: S3 Fig — (TIFF) [file pntd.0006807.s003.tiff]
