# Supplementary material for: Reactive and pre-emptive vaccination strategies to control hepatitis E infection in emergency and refugee settings: A modelling study
Source: PLoS Negl Trop Dis. 2018 Sep 25;12(9):e0006807. doi: 10.1371/journal.pntd.0006807 (PMC6173446; doi:10.1371/journal.pntd.0006807)
Supplement: S1 Appendix — (PDF) [file pntd.0006807.s004.pdf]

S1 Appendix: Model equations

Models were implemented as a set of ordinary differential equations. These are given in Equations 1 below for Models 1 and 2 with additional terms to account for the effect of vaccination (not used in model fitting as no vaccine was used, but used in the evaluation of potential vaccination campaigns). Equations 2 represent Model 6, again with extensions to account for vaccination.

Model 1(the baseline model) corresponds to an SEIR model. In sensitivity analyses variations of this model are used making different assumptions about the distributions of latent and infectious periods (Models 3-5), relaxing the assumption of constant transmissibility when infectious (Models 2 and 5) and explicitly accounting for a saturating environmental reservoir (Model 6). Equations for Models 3-5 are not shown but these are simple extensions of Model 1, where in Model 3 the rate of leaving compartment E_1 was the same as the rate of leaving E_2 and in Models 4 and 5 the rate of leaving compartment I_1 was the same as the rate of leaving I_2. Model 5 differed from Model 4 in that one extra parameter was estimated: the proportion of transmission events that resulted from contacts with patients in the I_1 compartment.

Equations 1:

Equations governing the epidemic in a single camp (allowing for vaccination) in Models 1 and 2 are:

$$\frac{dS_{NV}}{dt} = -\frac{\beta(t)S_{NV}(I_{NV}+I_V)}{N}$$

$$\frac{dS_V}{dt} = -\frac{\beta(t)S_V(I_{NV}+I_V)}{N} - \theta S_V \sum_{t_v \in \{\tau_2,\tau_3 \ldots\}} \delta(t-t_v)$$

$$\frac{dE_{NV}}{dt} = \frac{\beta(t)S_{NV}(I_{NV}+I_V)}{N} - \gamma E_{NV}$$

$$\frac{dE_V}{dt} = \frac{\beta(t)(V_S+S_V)(I_{NV}+I_V)}{N} - \gamma E_V$$

$$\frac{dI_{NV}}{dt} = \gamma E_{NV} - \rho I_{NV}$$

$$\frac{dI_V}{dt} = \gamma E_V - \rho I_V$$

$$\frac{dR_{NV}}{dt} = \rho I_{NV}$$

$$\frac{dR_V}{dt} = \rho I_V + (\xi_2\theta S_V + \xi_3\theta V_S) \sum_{t_v \in \{\tau_2,\tau_3 \ldots\}} \delta(t-t_v)$$

$$\frac{dV_S}{dt} = -\frac{\beta(t)V_S(I_{NV}+I_V)}{N} + ((1-\xi_2)\theta S_V - \xi_3\theta V_S) \sum_{t_v \in \{\tau_2,\tau_3 \ldots\}} \delta(t-t_v)$$

Where the variables and parameters are:

S_{NV}: Susceptible, but not in the group covered by the vaccination policy
S_V: Susceptible, and in the group covered by the vaccination policy but not yet vaccinated
E_{NV}: Exposed (infected but not yet infectious), but not in the group covered by the vaccination policy
E_V: Exposed (infected but not yet infectious), in the group covered by the vaccination policy
I_{NV}: Infected and infectious, but not in the group covered by the vaccination policy
I_V: Infected and infectious, in the group covered by the vaccination policy
R_{NV}: Removed (and immune), but not in the group covered by the vaccination policy
R_V: Removed (and immune), in the group covered by the vaccination policy

V_S: Vaccinated (with two doses) but still susceptible
$\beta(t)$: Transmission parameter which is proportional to the rate of transmission from one infectious person to one particular susceptible person at time *t.*
γ : Rate of progression from exposed to infectious compartment
ρ: Rate of progression from the infectious to the immune compartment
θ: The proportion of those in the group eligible for vaccination who receive the vaccine
ξ_2: The proportion of those who have been vaccinated with two doses who are successfully immunized as a result.
ξ_3: The proportion of those remaining susceptible after being vaccinated with two doses who are successfully immunized as a result of a third vaccine dose.

In Model 1 $\beta(t)$ was taken as a constant. In Model 2a $\beta(t)$ was a step function taking the value β_0 in time periods before initiation of the water and sanitation intervention and β_1 afterwards. In Model 2b $\beta(t)$ was allowed to depend on the water sources per person data according to the relationship $\beta(t) = \beta_0 exp(\omega\, w(t))$ where w(t) is the interpolated number of water sources per person (S1 Figure), and ω the model parameter to be estimated. Values of ω less than zero would indicate reduced transmission as more water sources per person are available, values greater than zero would indicate increased transmission, and values equal to zero correspond to no effect.

The term $\delta(x)$ is the Dirac delta function (equal to zero unless its argument is zero). Vaccination is assumed to generate immunity instantaneously at times τ_2 , τ_3 (assumed to be two weeks after the second and third doses of the vaccine).

Since there is no evidence of any effect of a single vaccine dose, we conservatively assume it has no effect. We model immunity arising from vaccination by assuming that immunity is generated as a result of two or three doses. A proportion θ of individuals eligible to receive the vaccine under given scenario assumptions are assumed to receive the first two doses of vaccine at a time such that immunity is generated at time τ_2 (following the second dose). In all vaccine scenarios we set θ to 0.9, which is consistent with vaccine coverage levels achieved in other refugee camp settings. The probability that such immunity is generated as a result of two doses is ξ_2. No immunity is assumed to be generated as a result of vaccination prior to this time. Those who do generate immunity as a result of vaccination are assumed to develop full protection (with no subsequent loss of immunity). In scenarios where we consider pre-emptive vaccination with three doses, for those who receive two doses but remain susceptible, a proportion, θ, are assumed to receive the third dose and the probability of generating immunity as a result is ξ_3. The value of ξ_3 is chosen to ensure that the probability that someone who has received three vaccine doses has immunity corresponds to the 3-dose vaccine effectiveness estimate, VE_3, which is sampled from the posterior distribution (see S3 Vaccine effectiveness). Since $VE_3 = \xi_2 + (1-\xi_2)\,\xi_3$ we have $\xi_3 = \frac{VE_3-\xi_2}{1-\xi_2}$.

Equations governing the epidemic in a single camp allowing for vaccination and a saturating environmental reservoir (Model 6) are:

Equations 2:

$$\frac{dS_{NV}}{dt} = -\frac{\beta(t)S_{NV}(I_{NV}+I_V)}{N} - \frac{\beta' W S_{NV}}{N(1+W)}$$

$$\frac{dS_V}{dt} = -\frac{\beta(t)S_V(I_{NV}+I_V)}{N} - \frac{\beta' W S_V}{N(1+W)} - \theta S_V \sum_{t_v \in \{\tau_2,\tau_3 \ldots\}} \delta(t-t_v)$$

$$\frac{dE_{NV}}{dt} = \frac{\beta(t)S_{NV}(I_{NV}+I_V)}{N} + \frac{\beta' W S_{NV}}{N(1+W)} - \gamma E_{NV}$$

$$\frac{dE_V}{dt} = \frac{\beta(t)(V_S+S_V)(I_{NV}+I_V)}{N} + \frac{\beta' W S_V}{N(1+W)} - \gamma E_V$$

$$\frac{dI_{NV}}{dt} = \gamma E_{NV} - \rho I_{NV}$$

$$\frac{dI_V}{dt} = \gamma E_V - \rho I_V$$

$$\frac{dR_{NV}}{dt} = \rho I_{NV}$$

$$\frac{dR_V}{dt} = \rho I_V + (\xi_2 \theta S_V + \xi_3 \theta V_S) \sum_{t_v \in \{\tau_2, \tau_3 \ldots\}} \delta(t - t_v)$$

$$\frac{dV_S}{dt} = -\frac{\beta(t) V_S (I_{NV} + I_V)}{N} + ((1 - \xi_2)\theta S_V - \xi_3 \theta V_S) \sum_{t_v \in \{\tau_2, \tau_3 \ldots\}} \delta(t - t_v)$$

$$\frac{dW}{dt} = \lambda (I_V + I_{NV}) - \upsilon W$$

Where

W: is a measure of contamination in the saturating environmental reservoir.
β': is a parameter to account for the rate of transmission from the environmental reservoir;

λ is the rate of increase in the contamination of this environment per infected host;
υ is the rate of loss of contamination from this reservoir.

Other variables and parameters are the same as in Equations 1.

When fitting this model to data, we place a prior on the proportion, p, of secondary cases per case arising via the environmental reservoir at the start of the epidemic.

Thus $p = R_0^E / (R_0^E + R_0^D)$, where R_0^E is the contribution to the basic reproduction number due to the environmental route, and R_0^D is the contribution due to the direct route. These are given by $R_0^D = \beta / \rho$ and $R_0^E = \beta' \lambda / (\rho \upsilon)$.
