# Supplementary material for: Reactive and pre-emptive vaccination strategies to control hepatitis E infection in emergency and refugee settings: A modelling study
Source: PLoS Negl Trop Dis. 2018 Sep 25;12(9):e0006807. doi: 10.1371/journal.pntd.0006807 (PMC6173446; doi:10.1371/journal.pntd.0006807)
Supplement: S2 Appendix — (PDF) [file pntd.0006807.s005.pdf]

**S2 Appendix: Vaccine effectiveness assumptions**

Table A below lists the numbers of patients included in vaccine and placebo arms and the number of infections for the analysis of the vaccine effectiveness for two and three doses. The two dose analysis included only patients who received doses 1 and 2 (regardless of whether they received dose 3) but excluded those in the reactogenicity subset because these lacked follow-up between 0 and 6 months following the first dose. For this analysis we used only the number of confirmed Hepatitis E infections occurring in these patients between months 1.5 and 6 following the first dose (after the patients received the second dose and before any had received the third dose). The three dose analysis included only patients who had received all three doses, and included Hepatitis E infections occurring in the follow-up period between 7 and 19 months following the first dose.

Posterior distribution for vaccine effectiveness after two and three doses were derived using source data from Zhu et al [23] using an approach corresponding to method E in Ewell[1]. The prior model assumed independent risks of infection in each arm that followed a Beta distribution

$$p(\omega) \propto \omega^{\alpha-1}(1-\omega)^{\beta-1}$$

where we took $\alpha = \beta = 1$ (which assigns each possible value for the risk an equal probability density). The posterior distributions for the risks of infection in each arm (which represent our uncertainty about these risks having observed the data) were derived assuming a binomial model for the observed data and using beta-binomial conjugacy. Thus if the data we have show that there were *n* people at risk in a particular arm, and *c* of these became infected, then the posterior distribution would be given by

$$p(\omega|\text{data}) \propto \omega^{\alpha+c-1}(1-\omega)^{\beta+n-c-1}.$$

If the risk of infection in the vaccine group is $r_v$ and the risk of infection in the placebo group is $r_p$, then vaccine effectiveness is given by $100 \times (1 - r_v / r_p)$. We estimated the posterior distribution of this statistic by simulation using 100,000 samples from the posterior distributions of $r_v$ and $r_p$ (Fig A, below).

This analysis gives posterior means (and central 95% credible intervals) of 80.2% (16.4%, 99.6%) for two doses and 93.3% (74.3%, 99.8%) for 3.

Table A: Source data for vaccine estimates of vaccine efficacy (from Zhu et al[1]).

| **Two dose analysis** | **Placebo group** | **Vaccine group** |
|---|---|---|
| Number of people included | 50838 | 50919 |
| Number of infections | 5 | 0 |
| **Three dose analysis** | | |
| Number of people included | 48663 | 48693 |
| Number of infections | 15 | 0 |

**Fig A. Probability distributions for two and three dose vaccine efficacy**

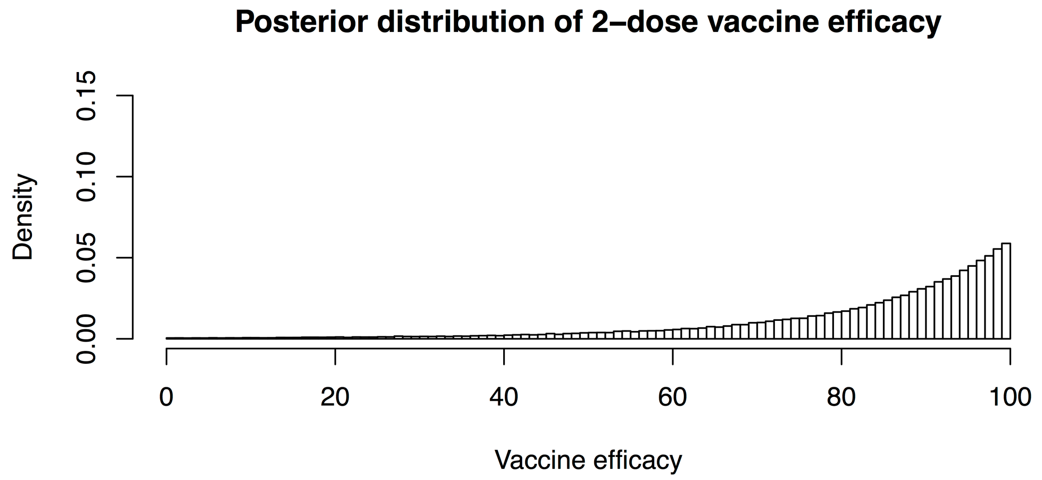


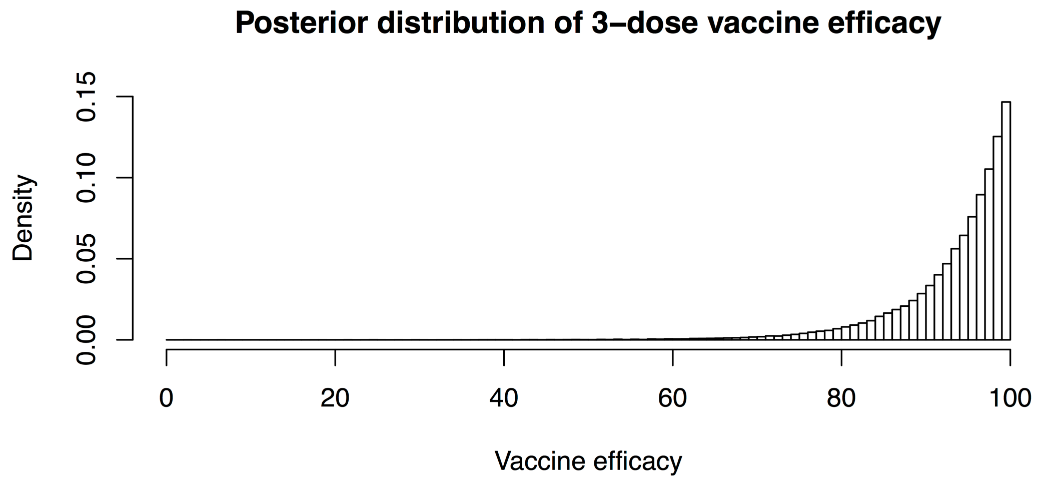


References:

[1] Ewell M. Comparing methods for calculating confidence intervals for vaccine efficacy. Stat Med.1996;15: 2379-2392.
