# Supplementary material for: Reactive and pre-emptive vaccination strategies to control hepatitis E infection in emergency and refugee settings: A modelling study
Source: PLoS Negl Trop Dis. 2018 Sep 25;12(9):e0006807. doi: 10.1371/journal.pntd.0006807 (PMC6173446; doi:10.1371/journal.pntd.0006807)

S3 Appendix: Plots of Markov chain Monte Carlo output from model 1

Fig A

Markov chains from the first 10, million iterations plotting every 100th iteration.

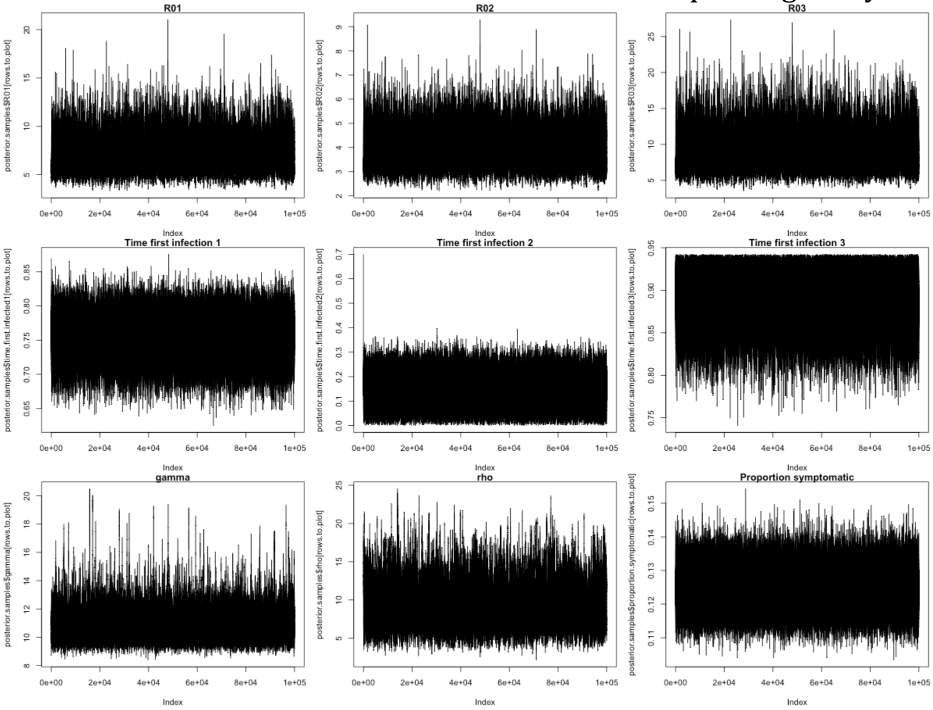

Fig B

Pairwise plots of the samples in the figure above to illustrate correlations.

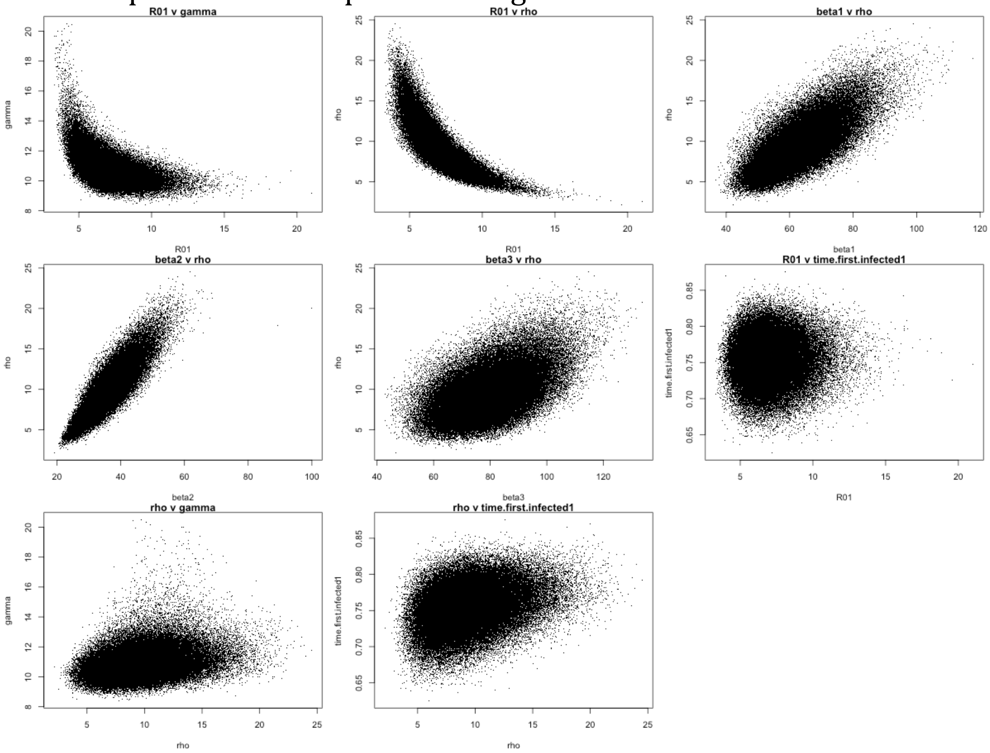

Supplement: S3 Appendix — (PDF) [file pntd.0006807.s006.pdf]
