# Supplementary material for: Reactive and pre-emptive vaccination strategies to control hepatitis E infection in emergency and refugee settings: A modelling study
Source: PLoS Negl Trop Dis. 2018 Sep 25;12(9):e0006807. doi: 10.1371/journal.pntd.0006807 (PMC6173446; doi:10.1371/journal.pntd.0006807)
Supplement: S1 Table — (PDF) [file pntd.0006807.s007.pdf]

Parameter	**Model 1: SEIR**<br><br>**Baseline assumption: population fully susceptible at start of outbreak**	**Model 1c: SEIR**<br><br>**Baseline model but with 10% of the population assumed to be initially immune**	**Model 1d: SEIR**<br><br>**Baseline model but with 20% of the population assumed to be initially immune**	**Model 1e: SEIR**<br><br>**Baseline model but with 30% of the population assumed to be initially immune**
Mean latent period (days)	34.4 (28.8, 38.8)	34.3 (28.5, 38.8)	34.2 (28.9, 38.8)	34.2 (28.8, 38.8)
Mean infectious period (days)	35.9 (20.9, 64.4)	36.0 (21.4, 65.2)	35.6 (21.5, 64.6)	38.3 (21.6, 64.8)
Infections reported (%)	12.5 (11.4, 13.6)	13.8 (12.7, 15.1)	15.6 (14.3, 17.0)	17.8 (16.3, 19.4)
R_0 Agoro	6.5 (4.5, 9.9)	7.2 (5.0, 11.0)	8.4 (5.7, 12.4)	9.6 (6.5, 14.2)
R_0 Madi Opei	3.7 (2.8, 5.1)	4.2 (3.0, 5.7)	4.7 (3.5, 6.4)	5.4 (4.0, 7.3)
R_0 Paloga	8.5 (5.3,11.4)	9.4 (5.9, 14.7)	10.5 (6.7, 16.5)	12.1 (7.6, 12.9)
Day 1st infection Agoro	278 (257, 298)	280 (260, 300)	283 (263, 302)	286 (265, 304)
Day 1st infection Madi Opei	62 (16, 103)	65 (19, 106)	70 (24, 110)	74 (28, 114)
Day 1st infection Paloga	327 (305, 343)	328 (307, 343)	330 (310, 343)	331 (312, 343)
